# Prevalence and pattern of dyslipidemia in patients with type 2 diabetes mellitus in Zaria, Northwestern Nigeria

**DOI:** 10.11604/pamj.2019.34.123.18717

**Published:** 2019-10-31

**Authors:** Beatrice Ohunene Bello-Ovosi, Joseph Ogirima Ovosi, Modupe Arinola Ogunsina, Sunday Asuke, Muhammed Sani Ibrahim

**Affiliations:** 1 Department of Internal Medicine, Kaduna State University, Barau Dikko Teaching Hospital, Kaduna, Nigeria; 2 461 Nigerian Air Force Hospital, Kaduna, Nigeria; 3 Department of Community Medicine, Bingham University, Jos, Nigeria; 4 Department of Community Medicine, Ahmadu Bello University, Zaria, Kaduna, Nigeria

**Keywords:** Prevalence, dyslipidemia, diabetes mellitus, Nigeria

## Abstract

**Introduction:**

Dyslipidemia confers excess atherosclerotic cardiovascular risk in type 2 diabetes mellitus (DM) patients, and this requires prompt identification and management to reduce morbidity and mortality. This study assessed the prevalence and pattern of dyslipidemia in type 2 DM patients in Zaria, Northwestern Nigeria.

**Methods:**

This was a cross-sectional study of newly diagnosed type 2 DM patients at Ahmadu Bello University Teaching Hospital (ABUTH), Zaria. Demographic, clinical and laboratory data were extracted from the case notes of eligible patients and analyzed using STATA version 14. Continuous variables were presented as mean ± standard deviation (SD), or median and interquartile range (IQR) while categorical variables were as frequencies and percentages. Student t and chi-square tests were used to test for association at p < 0.05.

**Results:**

A total of 322 subjects (161 male, 161 female) with a mean age of 53.5 ± 10.8 years partook in the study. The prevalence of dyslipidemia was 69.3%. Mixed dyslipidemia of high triglyceride (TG) and high low-density lipoprotein cholesterol (LDL-C) was present in 41.0%; high TG and low high-density lipoprotein cholesterol (HDL-C) in 2.8%; and high LDL and low HDL in 2.5%. Atherogenic dyslipidemia, isolated hypercholesterolemia and isolated low HDL-cholesterol were present in 3.4%, 2.5% and 23.6% respectively. Dyslipidemia status was not associated with age, sex, duration of DM or hypertension, obesity, and mean fasting blood sugar (FBS) and 2-hour postprandial glucose.

**Conclusion:**

The prevalence of dyslipidemia is high in the newly diagnosed type 2 DM patients and therefore, initial management should incorporate measures to control dyslipidemia.

## Introduction

Atherosclerotic cardiovascular diseases (ASCVD) defined as coronary heart disease, cerebrovascular disease, or peripheral arterial disease presumed to be of atherosclerotic origin represents the largest proportion of cardiovascular diseases (CVD), and is a leading cause of premature mortality and disability-adjusted life years (DALYs) globally [1-3]. In 2015, cardiovascular diseases were responsible for 31% (17.92 million) of the global annual deaths; out of which coronary heart disease (CHD) and cerebrovascular disease (stroke) claimed 7.4 million and 6.7 million lives respectively; and more than 75% of these occur in low- and middle-income countries [4,5]. Of the several risk factors attributed to the rising prevalence of atherosclerotic cardiovascular diseases, diabetes mellitus (DM) and dyslipidemia stand out as the most preventable and modifiable [6-8]. Nevertheless, rapid urbanization accompanied with changing lifestyle and demographics have led to a surge in the global prevalence of these twin diseases. In 2017, DM affected 425 million people aged 20-79 years globally, and this is projected to reach 629 million by 2045; 90% of this will be type 2 DM, and 79% will be from low- and middle-income countries [9]. Also, in 2008, raised cholesterol level ≥ 5.0 mmol/L occurred in 39% of the global adult population, 22.6% of this occurs in the African sub-region [10]. DM and dyslipidemia are intricately related and their co-existence helps to perpetuate ASCVD. DM confers at least two to threefold excess risk, independent of other risk factors for ASCVD [11-13]; and patients with DM have poorer prognosis after cardiovascular events compared to non-diabetic patients [14]. On the other hand, the risk of CVD is greater at any level of serum cholesterol in patients with diabetes [15], and several studies have shown that cholesterol lowering therapies reduce the incidence of cardiovascular diseases in both diabetic and non-diabetic patients [16-18]. In type 2 DM, insulin deficiency and/or resistance trigger a cascade of metabolic disarray leading to characteristic lipoproteins abnormality termed atherogenic (diabetic) dyslipidemia, defined as: low levels of high-density lipoprotein cholesterol (HDL-C), elevated triglycerides (TG) and low-density lipoprotein cholesterol (LDL-C) [19]. In this study, we determined the prevalence and pattern of diabetic dyslipidemia in type 2 DM patients in Zaria Northern Nigeria.

## Methods

**Study area:** this study was carried out at the Ahmadu Bello University Teaching Hospital (ABUTH), Zaria, Northwestern Nigeria. ABUTH is a 500-bed capacity hospital that provides services to patients from Northern Nigeria and the neighboring countries of Niger and Chad Republics.

**Study design:** this was a cross-sectional study of newly diagnosed type 2 DM patients who attended the Endocrine and Metabolism Clinic of ABUTH from 1^st^ January 2016 to 31^st^ December 2017.

**Study population:** subjects were newly diagnosed type 2 DM patients aged 30 years and above. Patients with gestational DM, steroid-induced DM, thyroid disease or secondary dyslipidemia were excluded from the study.

**Data collection:** demographic, clinical and laboratory data were retrieved from the case notes of patients who satisfied the eligibility criteria. Demographic data retrieved were age and sex, while clinical and laboratory data were: height, weight, waist circumference, duration of DM, blood pressure, duration of hypertension, fasting and 2-hour postprandial blood sugar over the last 3 months, fasting cholesterol level, fasting triglyceride level, fasting low density lipoprotein, fasting high density lipoprotein.

### Definition of variables

**Dyslipidemia:** this was defined as serum total cholesterol level ≥ 5.2mmol/L and/or serum LDL cholesterol ≥ 2.6 mmol/L, and/or serum triglyceride ≥ 1.7 mmol/L, and/or serum HDL cholesterol < 1mmol/L for men or < 1.3mmol/L for women [20]. Atherogenic dyslipidemia was defined as a combination of high serum triglyceride ≥ 1.7 mmol/L, high serum LDL cholesterol ≥ 2.6 mmol/L and low serum HDL cholesterol < 1mmol/L for men and < 1.30 mmol/L for women [21]. Non-HDL cholesterol ≥ 3.37mmol/L [22], and atherogenic index ≥ 0.11 [23,24], were also considered abnormal. Mixed dyslipidemia were defined as a combination of any of the following: high TG, low LDL; high TG, high LDL; high LDL, low HDL; and isolated dyslipidemia were defined as: isolated hypercholesterolaemia - combination of high TC and normal/low TG and LDL; isolated hypertriglyceridemia - combination of high TG and normal/low TC and LDL; isolated high LDL - combination high LDL and normal/low TG, TC while isolated low HDL was defined as combination of low HDL with normal LDL, TG and TC.

**Hypertension:** hypertension was defined as a blood pressure recording of ≥ 140/90 mmHg on more than 1 hospital visit or a documentation of treatment with anti-hypertensive medications [25].

**Type 2 diabetes mellitus:** this was defined as documentation of fasting blood sugar ≥ 7.0 mmol/L or 2h postprandial blood sugar ≥ 11.1 mmol/L for the first time in a patient, with or without classical symptoms of DM; or presentation for the first time with symptoms of hyperglycemic crises and a documented random blood sugar ≥ 11.1 mmol/L; and good glycemic target was defined as preprandial capillary plasma glucose between 4.4-7.2 mmol/L and 2h postprandial capillary plasma glucose < 10.0 mmol/L [26].

**Anthropometry:** body mass index was classified as: normal (BMI 18.5-24.9), overweight (BMI 25.0-29.9), obesity (BMI ≥ 30) [27]. Truncal obesity was defined as waist circumference > 94 cm for males and > 80 cm for females [28].

**Statistical analysis:** data were coded and entered into STATA version 14 (Stata Corp, College Station, Texas) for analysis. Continuous variables with symmetrical distribution were expressed as means ± standard, while those with skewed distribution were expressed as median and interquartile range. Categorical variables were expressed as frequencies and percentages. Student t-test and chi-square test were used to test for association. Statistical significance was set at p < 0.05.

**Ethical approval:** ethical approval for the research and permission for use of data was obtained from the Ahmadu Bello University Teaching Hospital Research Ethics Committee (ABUTH-REC). To maintain privacy and confidentiality, each patient was assigned a unique numerical identifier for tracking purposes only and data was retrieved anonymously.

## Results

A total of 322 patients (161 male and 161 female) with type 2 DM fulfilled the eligibility criteria and were included in the study. Their mean age was 53.5 ± 10.8 years while their mean body mass index and mean waist circumference were 27.8 ± 6.4 kg/m^2^ and 92.8 ± 13.1 cm respectively. The mean fasting blood sugar and mean 2-hour postprandial sugar were 7.9 ± 3.1 mmol/L and 10.7 ± 3.5 mmol/L respectively, and 227 (70.5%) of them were hypertensive. Other clinical characteristic such as mean fasting blood sugar, mean 2h postprandial blood sugar, median duration of diabetic mellitus, median duration of hypertension, and mean serum lipid levels of the patients are as shown in Table 1. The prevalence of dyslipidemia in the patients was 69.3%. There was no significant difference in the prevalence of dyslipidemia between the female and male sexes, 68.3% vs. 70.2%, p = 0.717 (difference in proportion = -0.02, 95% CI = -0.12 to -0.08). The prevalence of atherogenic dyslipidemia, isolated hypercholesterolemia, isolated low HDL-cholesterol were 3.4%, 2.5%, and 23.6% respectively. Isolated hypertriglyceridemia and isolated high LDL cholesterol were not seen in these patients. The prevalence of mixed dyslipidemias were: high TG and low HDL (2.8%), high TG and high LDL (41.0%) and high LDL and low HDL (2.5%). There were no statistically significant differences in the prevalence of the various dyslipidemia between the sex groups (Table 2). Table 3 shows dyslipidemia status by demographic and clinical characteristics of the subjects. There were no statistically significant differences in the trend of dyslipidemia across age groups, duration of DM, duration of hypertension and grade of obesity. There were also no statistically significant differences in the proportion of males, hypertensives, truncal obesity, target mean FBS and target mean 2h postprandial sugar between subjects who had dyslipidemia and those with normolipemia.

**Table 1:** Demographic and clinical characteristics of patients studied

Parameters	Results
**Age (years), mean ± SD**	**53.5 ± 10.8**
30-44	62 (19.2)
45-59	159 (49.4)
60-74	93 (28.9)
≥ 75	8 (2.5)
**Sex, n (%)**	
Male	161 (50.0)
Female	161 (50.0)
**Body Mass Index (kg/m^2^), mean ± SD**	**27.8 ± 6.4**
18.5-24.9 (Normal)	112 (34.9)
25.0-29.9 (Overweight)	104 (32.4)
30.0-34.9 (Grade 1 obesity)	62 (19.1)
35.0-39.9 (Grade 2 obesity)	30 (9.2)
≥ 40.0 (Morbid obesity)	14 (4.4)
**Waist circumference (cm), mean ± SD**	**92.8 ± 13.1**
< 80 (women), < 94 (men)	122 (37.9)
≥ 80(women), ≥ 94 (men)	200 (62.1)
**Mean FBS (mmol/L), mean ± SD**	**7.9 ± 3.1**
< 7.2	160 (49.7)
≥ 7.2	162 (50.3)
**Mean 2hPP (mmol/L), mean ± SD**	**10.7 ± 3.5**
< 10.0	142 (44.1)
≥ 10.0	180 (55.9)
**Duration of DM (years); median, IQR**	**4.3 (2, 8)**
< 1	32 (9.9)
1-5	162 (50.3)
6-9	80 (24.9)
≥10	48 (14.9)
**Co-existing hypertension, n (%)**	
No	95 (29.5)
Yes	227 (70.5)
**Duration of hypertension (years); median, IQR**	**5 (2, 9)**
< 1	13 (5.7)
1-5	125 (55.1)
6-9	50 (22.0)
≥10	39 (17.2)
**Total Cholesterol (mmol/L), mean ± SD**	**5.1 ±1.2**
< 5.2	158 (49.2)
≥ 5.2	164 (50.8)
**Triglyceride (mmol/L), mean ± SD**	**1.6 ± 0.8**
< 1.7	190 (58.9)
≥ 1.7	132 (41.1)
**High-density lipoprotein (mmol/L), mean ± SD**	**1.4 ± 0.7**
< 1.03 (men), < 1.30 (women	115 (35.6)
≥ 1.03 (men), ≥ 1.30 (women)	207 (64.4)
**Low -density lipoprotein (mmol/L), mean ± SD**	**2.7 ± 1.30**
< 2.6	148 (46.1)
≥ 2.6	174 (53.9)
**Non-High-density lipoprotein (mmol/L), mean ± SD**	**3.7 ± 1.2**
< 3.37	110 (34.3)
≥ 3.37	212 (65.7)
**Atherogenic Index, mean ± SD**	**0.16 ± 0.65**
< 0.11	159 (49.4)
≥ 0.11	163 (50.6)

SD = Standard Deviation, IQR = Interquartile Range, FBS = Fasting Blood Sugar, 2h PP = 2 hour Postprandial *

**Table 2:** Prevalence and pattern of dyslipidemia by sex of patients studied

Lipid abnormality	All, N (%)	Male, n (%)	Female, n (%)	P−value
**Dyslipidemia**				
High TC, and/or High LDL, and/or High TG, and/or Low HDL	223 (69.3)	113 (70.2)	110 (68.3)	0.717
**Atherogenic dyslipidemia**				
High TG, High LDL, and Low HDL	11 (3.4)	6 (3.7)	5 (3.1)	0.521
**Isolated dyslipidemias**				
High TC	8 (2.5)	3 (1.9)	5 (3.1)	0.401
High TG	0 (0)	0 (0)	0 (0)	
High LDL	0 (0)	0 (0)	0 (0)	
Low HDL	76 (23.6)	40 (24.8)	36 (22.3)	0.621
**Mixed dyslipidemias**				
High TG and low LDL	9 (2.8)	5 (3.1)	4 (2.5)	0.742
High TG and High LDL	132 (41.0)	67 (41.6)	65 (40.4)	0.892
High LDL and low HDL	8 (2.5)	5 (3.1)	3 (1.9)	0.485

TC = Total Cholesterol, LDL = Low-density lipoprotein, TG = Triglyceride, HDL = High-density lipoprotein

**Table 3:** Dyslipidemia status by demographic and clinical characteristics of patients studied

Variables	Dyslipidemia, n (%)	Normolipemia, n (%)	P−value
**Age, years**			0.230
30-44	40 (17.9)	22 (22.2)	
45-59	109 (48.9)	50 (50.5)	
60-74	68 (30.5)	25 (25.3)	
≥ 75	6 (2.7)	2 (2.0)	
**Sex, n (%)**			0.717
Male	113 (50.7)	51 (48.5)	
Female	110 (49.3)	48 (51.5)	
**Duration of DM, years**			0.461
< 1	20 (9.0)	12 (12.1)	
1-5	115 (51.6)	47 (47.5)	
6-9	51 (22.9)	29 (29.3)	
> 10	37 (16.6)	11 (11.1)	
**Hypertensive, n (%)**			0.072
No	59 (26.5)	36 (36.4)	
Yes	164 (73.5)	63 (63.6)	
**Duration of hypertension, years**			0.081
< 1	10 (6.2)	3 (4.8)	
1-5	86 (52.4)	39 (61.9)	
6-9	33 (20.1)	17 (27.0)	
> 10	35 (21.3)	4 (6.3)	
**BMI, kg/m^2^**			0.269
Normal (18.5 – 24.9)	75 (34.3)	35 (36.5)	
Overweight (25.02 – 9.9)	69 (31.5)	33 (34.4)	
Grade 1 obesity (30.0 – 34.9)	42 (19.2)	18 (18.8)	
Grade 2 obesity (35.0 – 39.9)	21 (9.6)	8 (8.3)	
Morbid obesity (≥ 40.0)	12 (5.4)	2 (2.0)	
**Waist circumference, cm**			0.384
Normal	89 (39.6)	35 (34.4)	
Truncal obesity	134 (60.5)	64 (65.6)	
**Mean FBS, mmol/L**			0.834
< 7.2 mmol/L	112 (50.2)	47 (48.9)	
≥ 7.2 mmol/L	111 (49.8)	49 (51.1)	
**Mean 2hPP, mmol/L**			
< 10.0 mmol/L	103 (46.0)	39 (39.8)	0.313
≥ 10.0 mmol/L	120 (54.0)	60 (60.2)	

FBS = Fasting Blood Sugar, 2hPP = 2h Postprandial, BMI = Body Mass Index

## Discussion

Diabetic dyslipidemia confers at least two-to-threefold excess risk for premature atherosclerosis of large- and medium-sized vessels, independent of other risk factors; and numerous large-scale epidemiological studies and well-controlled clinical trials have shown a well-established association between lipid disorders and cardiovascular risks [29-32]. Dyslipidemia is an important modifiable risk factor for ASCVD and therefore, requires screening and treatment as a public health priority. In this study, the prevalence of dyslipidemia in type 2 diabetes mellitus patients was 69.3%. This is consistent with a prevalence of 34.4-94.0% reported from several studies across the globe [33-39]. Studies in Nigeria have also reported a prevalence ranging from 70% in Ibadan [40], to 89.0% in Lagos [41], and 90.7% in Nnewi [42]. This divergent range of global and regional prevalence of dyslipidemia among type 2 DM is attributed to the varying dyslipidemia cut-off thresholds used in most of the studies. While some studies use the National Cholesterol Education Program- Adult Treatment Panel III (NCEP-ATP III) threshold for dyslipidemia with a tighter cut-off [20], others used the WHO dyslipidemia threshold [43]. One thing that is more consistent is the fact that the prevalence of dyslipidemia among the diabetic population is much higher than reported in the epidemiological studies involving non-diabetic patients in Nigeria [44].

Atherogenic dyslipidemia of 3.4% in our patients signifies an increase in apolipoprotein B (ApoB), and a shift of LDL pool towards small, dense LDL that are cholesterol-ester depleted, and more atherogenic with higher risk for ASCVD [45,46]. Isolated hypercholesterolemia and isolated low LDL were seen in only 2.5% and 23.6% of the patients respectively, but studies have shown that apparently normal cholesterol level may mask the qualitative alteration in composite lipoprotein particles such as intermediate-density lipoprotein (IDL), small LDL and ApoB which are not routinely measured in clinical practice but which are nonetheless better predictors of risk of ASCVD [47]. There were no cases of isolated high LDL and isolated hypertriglyceridemia in this study, a finding suggestive of the probable multifactorial nature of diabetic dyslipidemia [48]. Mixed dyslipidemia is characteristic of typical type 2 DM patients [49] and in this study, high TG and low HDL; high TG and high LDL; and high LDL and low HDL were present in 2.8%, 41.0% and 2.5% of the patients respectively. Data from the United Kingdom Prospective Diabetes Study (UKPDS) have shown that patients with type 2 DM had higher TG and lower HDL cholesterol compared with the nondiabetics [30] and patients with co-occurrence of high TG and low HDL are at increased risk of major coronary events [50,51]. The prevalence and pattern of dyslipidemia in the patients were not affected by sex, age, duration of diabetes, hypertensive state, duration of hypertension, body mass index, truncal obesity and mean FBS and 2hPP glucose. Though these findings are in agreement with findings from Omotoye *et al.* [40] and Sang *et al.* [37]; findings from the UKPDS [30], Goel *et al.* [52], and Pokharel *et al.* [38] show varying association of dyslipidemia with some sociodemographic and clinical parameters of type 2 DM patients.

## Conclusion

The study highlights the high prevalence of dyslipidemia in patients with type 2 diabetes mellitus attending clinic at ABUTH, Zaria, Nigeria with mixed dyslipidemia of TG and high LDL being the commonest pattern. This implies that many of the type 2 DM patients are at a higher risk of ASCVD. There is, therefore, the need to prioritize a comprehensive lipid care in the management of all type 2 DM patients.

### What is known about this topic

- Diabetes mellitus and dyslipidemia are independent risk factors for atherosclerotic cardiovascular disease;
- Dylipidemia confers excess risk of adverse cardiovascular event in diabetes mellitus patients;
- Lipid management reduces cardiovascular events in both diabetic and non-diabetic patients.

### What this study adds

- The prevalence of dyslipidemia in type 2 DM patients in Zaria is 69.3%;
- Mixed dyslipidemia of high triglyceride and high low-density lipoprotein was the predominant pattern of dyslipidemia.

## Competing interests

The authors declare no competing interests.

## References

[1] American Diabetic Association (2018). Cardiovascular disease and risk management: Standards of Medical Care in Diabetes-2018. Diabetes Care.

[2] Reiner Z, Catapano AL, De Backer G, Graham I, Taskinen MR, European Association for Cardiovascular Prevention & Rehabilitation (2011). ESC/EAS Guidelines for the management of dyslipidaemias: the Task Force for the management of dyslipidaemias of the European Society of Cardiology (ESC) and the European Atherosclerosis Society (EAS). Eur Heart J.

[3] Allender S, Scarborough P, Peto V, Rayner M, Leal J, Luengo-Fernandez R, Gray A (2008). European Cardiovascular disease statistics 2008 edition.

[4] Roth GA, Johnson C, Abajobir A, Abd-Allah F, Abera SF, Abyu G (2017). Global, Regional, and National Burden of Cardiovascular Diseases for 10 Causes, 1990 to 2015. J Am Coll Cardiol.

[5] World Health Organization Global Heart Initiative: The Global cardiovascular disease (CVD).

[6] Dawber TR (1980). The Framingham Study: The epidemiology of atherosclerotic disease.

[7] Mendis S (2010). The contribution of the Framingham Heart Study to the prevention of cardiovascular disease: A global perspective. Prog Cardiovasc Dis.

[8] Keys A (1980). Seven countries: A multivariate analysis of death and coronary heart disease.

[9] International Diabetes Federation (2017). IDF Diabetes Atlas.

[10] World Health Organization Global Health Observatory data: Cholesterol.

[11] Levitan EB, Song Y, Ford ES, Lui S (2004). Is non-diabetic hyperglycaemia a risk factor for cardiovascular disease? A meta-analysis of prospective studies. Arch Intern Med.

[12] Eberly LE, Cohen JD, Prineas R, Yang L, Intervention Trial Research group (2003). Impact of incident diabetes and incident nonfatal cardiovascular disease on 18-year mortality: The multiple risk factor intervention trial experience. Diabetes Care.

[13] Kannel WB, McGee DL (1979). Diabetes and cardiovascular disease: A Framingham study. JAMA.

[14] Laing SP, Swerdlow AJ, Slater SD, Burden AC, Morris A, Waugh NR (2003). Mortality from heart disease in a cohort of 23,000 patients with insulin-treated diabetes. Diabetologia.

[15] Stamler J, Vaccaro O, Neaton JD, Wentworth D (1993). Diabetes, other risk factors, and 12-yr cardiovascular mortality for men screened in the Multiple Risk Factor Intervention Trial. Diabetes Care.

[16] Colhoun HM, Betteridge DJ, Durrington PN, Hitman GA, Neil HA, Livingstone SJ (2004). Primary prevention of cardiovascular disease with atorvastatin in type 2 diabetes in the collaborative atorvastatin diabetes study (CARDS): multicenter randomised placebo-controlled trial. Lancet.

[17] Baigent C, Keech A, Kearney PM, Blackwell L, Buck G, Pollicino C (2005). Efficacy and safety of cholesterol-lowering treatment: prospective meta-analysis of data from 90,056 participants in 14 randomised trials of statins. Lancet.

[18] Kearney PM, Blackwell L, Collins R, Keech A, Simes J, Cholesterol Treatment Trialists' (CTT) Collaborators (2008). Efficacy of cholesterol-lowering therapy in 18,686 people with diabetes in 14 randomised trials of statins: a meta-analysis. Lancet.

[19] Grundy SM (1997). Small LDL, Atherogenic dyslipidemia, and metabolic syndrome. Circulation.

[20] Expert Panel on Detection, Evaluation, and Treatment of High Blood Cholesterol in Adults (2001). Executive Summary of The Third Report of The National Cholesterol Education Program (NCEP) Expert Panel on Detection, Evaluation, And Treatment of High Blood Cholesterol In Adults (Adult Treatment Panel III). JAMA.

[21] Austin MA, King MC, Vranizan KM, Krauss RM (1990). Atherogenic lipoprotein phenotype. A proposed genetic marker forcoronary heart disease risk. Circulation.

[22] Jellinger PS, Handelsman Y, Rosenblit PD, Bloomgarden ZT, Fonseca VA, Garber AJ (2017). American association of clinical endocrinologists and American college of endocrinology guidelines for management of dyslipidemia and prevention of cardiovascular disease. Endocrine Pract.

[23] Dobiášová M, Frohlich J, Šedová M, Cheung MC, Brown BG (2011). Cholesterol esterification and atherogenic index of plasma correlate with lipoprotein size and findings on coronary angiography. J Lipid Res.

[24] Dobiášová M (2006). AIP--atherogenic index of plasma as a significant predictor of cardiovascular risk: from research to practice. Vnitr Lek.

[25] World Health Organization (2013). A global brief on hypertension.

[26] American Diabetes Association (2016). Standards of Medical Care in Diabetes-2016. Diabetes Care.

[27] World Health Organization (1995). Physical status: the use and interpretation of anthropometry.

[28] World Health Organization Waist circumference and waist-hip ratio: report of a WHO expert consultation.

[29] Chapman MJ (2006). Therapeutic elevation of HDL-cholesterol to prevent atherosclerosis and coronary heart disease. Pharmacol Ther.

[30] Turner RC, Millns H, Neil HA, Strattron IM, Manley SE, Matthews DR (1998). Risk factors for coronary artery disease in noninsulin dependent diabetes mellitus: United Kingdom Prospective Diabetes Study (UKPDS: 23). BMJ.

[31] Eberly LE, Stamler J, Neaton JD, Multiple Risk Factor Intervention Trial Research Group (2003). Relation of triglyceride levels, fasting and nonfasting, to fatal and nonfatal coronary heart disease. Arch Intern Med.

[32] Nordestgaard BG, Benn M, Schnohr P, Tybgaerd-Hansen A (2007). Nonfasting triglycerides and risk of myocardial infarction, ischemic heart disease, and death in men and women. JAMA.

[33] Manisha Mandal, Rina Kumari, Arati Mukherjee (2015). Prevalence of dyslipidemia in patients with type 2 diabetes mellitus: a hospital based study in Kishanganj, India. Int J Res Med Sci.

[34] Bekele S, Yohannes T, Mohammed AE (2017). Dyslipidemia and associated factors among diabetic patients attending Durame General Hospital in Southern Nations, Nationalities, and People's Region. Diabetes Met Syndr Obes.

[35] Razaq A, Mohammad T, Razaq A (2017). Prevalence of dyslipidemia in newly diagnosed type 2 diabetes patients at diagnosed at KGNTH Bannu. Gomal J Med Sci.

[36] Chamba NG, Shao ER, Sonda T, Lyaruu IA (2017). Lipid Profile of Type 2 Diabetic Patients at a Tertiary Hospital in Tanzania: Cross Sectional Study. J Endocrinol Diab.

[37] Sang VK, Kaduka L, Kamano J, Makworo D (2017). Prevalence of dyslipidemia and the associated factors among Type 2 diabetes patients in Turbo Sub-County, Kenya. J Endocrinol Diab.

[38] Pokharel DR, Khadka D, Sigdel M, Yadav NK, Achary S, Kafle R (2017). Prevalence and pattern of dyslipidemia in Nepalese individuals with type 2 diabetes. BMC Res Notes.

[39] Jan SS, Rehman A, Ahmad R, Khan TM, Ahmad A, Abrar A (2011). Evaluation of pattern of dyslipidemia in Type 2 diabetics in Swat. Gomal J of Med Sci.

[40] Omotoye FE, Fadupin GT (2017). Evaluation of Lipid Profile of Type 2 Diabetic Patients Attending an Urban Tertiary Health Facility in Nigeria. Indian J Nutri.

[41] Okafor CI, Fasanmade OA, Oke DA (2008). Pattern of dyslipidemia among Nigerians with type 2 DM. Niger J of Clinical Pract.

[42] Jisieike-Onuigbo NN, Unuigbe EI, Oguejiofor CO (2011). Dyslipidemias in type 2 diabetes mellitus patients in Nnewi South-East Nigeria. Ann Afr Med.

[43] World Health Organization (1994). Cardiovascular disease risk factors: new areas for research, report of a WHO scientific group.

[44] Oguejiofor OC, Onwukwe CH, Odenigbo CU (2012). Dyslipidemia in Nigeria: Prevalence and pattern. Ann Afr Med.

[45] Lamarche B, Tchernof A, Moorjani S, Cantin B, Dagenais GR, Lupien PJ (1997). Small, dense low-density lipoprotein particles as a predictor of the risk of ischemic heart disease in men - Prospective results from the Quebec Cardiovascular Study. Circulation.

[46] Berneis KK, Krauss RM (2002). Metabolic origins and clinical significance of LDL heterogeneity. J Lipid Res.

[47] Soran H, France MW, Kwok S, Dissanayake S, Charlton-Menys V, Younis NN (2011). Apolipoprotein B100 is a better treatment target than calculated LDL and non-HDL cholesterol in statin-treated patients. Ann Clin Biochem.

[48] Taskinen MR (2003). Diabetic dyslipidemia: from basic research to clinical practice. Diabetologia.

[49] Sugden M, Holness M (2011). Pathophysiology of diabetic dyslipidemia: implications for atherogenesis and treatment. Clinical Lipidology.

[50] Jeppesen J, Hein HO, Suadicani P, Gyntelberg F (1997). Relation of high TG-low HDL cholesterol and LDL cholesterol to the incidence of ischemic heart disease. An 8-year follow-up in the Copenhagen Male Study. Arterioscler Thromb Vasc Biol.

[51] Assman G, Schulte H (1992). Relation of high-density lipoprotein cholesterol and triglycerides to incidence of atherosclerotic coronary artery disease (the PROCAM experience). Prospective Cardiovascular Münster study. Am J Cardiol.

[52] Goel S, Garg PK, Malhotra V, Madan J, Mitra SK, Grover S (2016). Dyslipidemia in Type II Diabetes Mellitus - An assessment of the main lipoprotein abnormalities. Bangladesh Journal of Medical Science.

